# Parallel Evolution in Predatory *Bdellovibrio* sp. NC01 during Long-Term Coculture with a Single Prey Strain

**DOI:** 10.1128/aem.01776-22

**Published:** 2023-01-04

**Authors:** Kathryn Mulvey, Katherine Brosnan, Mackenzie Galvin, Sydney Mohr, Lauren Muldowney, Molly Oser, Laura E. Williams

**Affiliations:** a Department of Biology, Providence College, Providence, Rhode Island, USA; Koninklijk Nederlands Instituut voor Onderzoek der Zee

**Keywords:** experimental evolution, predation, predation efficiency, predatory bacteria, prey range, starvation survival

## Abstract

Experimental evolution provides a powerful tool for examining how *Bdellovibrio* evolves in response to unique selective pressures associated with its predatory lifestyle. We tested how *Bdellovibrio* sp. NC01 adapts to long-term coculture with Pseudomonas sp. NC02, which is less susceptible to predation compared to other Gram-negative bacteria. Analyzing six replicate *Bdellovibrio* populations across six time points spanning 40 passages and 2,880 h of coculture, we detected 30 to 40 new mutations in each population that exceeded a frequency of 5%. Nonsynonymous substitutions were the most abundant type of new mutation, followed by small indels and synonymous substitutions. After completing the final passage, we detected 20 high-frequency (>75%) mutations across all six evolved *Bdellovibrio* populations. Eighteen of these alter protein sequences, and most increased in frequency rapidly. Four genes acquired a high-frequency mutation in two or more evolved *Bdellovibrio* populations, reflecting parallel evolution and positive selection. The genes encode a sodium/phosphate cotransporter family protein (Bd2221), a metallophosphoesterase (Bd0054), a TonB family protein (Bd0396), and a hypothetical protein (Bd1601). Tested prey range and predation efficiency phenotypes did not differ significantly between evolved *Bdellovibrio* populations and the ancestor; however, all six evolved *Bdellovibrio* populations demonstrated enhanced starvation survival compared to the ancestor. These results suggest that, instead of evolving improved killing of Pseudomonas sp. NC02, *Bdellovibrio* evolved to better withstand nutrient limitation in the presence of this prey strain. The mutations identified here point to genes and functions that may be important for *Bdellovibrio* adaptation to the different selective pressures of long-term coculture with Pseudomonas.

**IMPORTANCE** *Bdellovibrio* attack and kill Gram-negative bacteria, including drug-resistant pathogens of animals and plants. This lifestyle is unusual among bacteria, and it imposes unique selective pressures on *Bdellovibrio*. Determining how *Bdellovibrio* evolve in response to these pressures is valuable for understanding the mechanisms that govern predation. We applied experimental evolution to test how *Bdellovibrio* sp. NC01 evolved in response to long-term coculture with a single Pseudomonas strain, which NC01 can kill, but with low efficiency. Our experimental design imposed different selective pressures on the predatory bacteria and tracked the evolutionary trajectories of replicate *Bdellovibrio* populations. Using genome sequencing, we identified *Bdellovibrio* genes that acquired high-frequency mutations in two or more populations. Using phenotype assays, we determined that evolved *Bdellovibrio* populations did not improve their ability to kill Pseudomonas, but rather are better able to survive starvation. Overall, our results point to functions that may be important for *Bdellovibrio* adaptation.

## INTRODUCTION

Because of their predatory lifestyle, *Bdellovibrio* species experience unique selective pressures compared to nonpredatory bacteria. How do *Bdellovibrio* evolve in response to these selective pressures? Experimental evolution offers a valuable and powerful tool to ask this question and explore the evolutionary trajectories of *Bdellovibrio* populations subjected to different selective pressures under controlled laboratory conditions. Given recent interest in predatory bacteria for biocontrol applications against drug-resistant bacterial infections (1, 2), experimental evolution enables researchers to manipulate and interrogate interactions between *Bdellovibrio* and prey bacteria and identify mechanisms that impact adaptation of *Bdellovibrio* to particular prey species or environmental conditions. Toward this end, we designed an experimental evolution scheme to test how *Bdellovibrio* evolves in response to long-term coculture with a single prey strain, using genome sequencing to identify mutations and phenotype assays to assess prey range and predation efficiency.

As the ancestor for experimental evolution, we used *Bdellovibrio* sp. NC01, which is an intraperiplasmic predator of Gram-negative bacteria. We previously isolated this strain from soil, sequenced its genome, and characterized its predatory phenotypes (3). The life cycle of NC01, along with other intraperiplasmic *Bdellovibrio*, involves two phases: attack phase and growth phase (2, 4). During attack phase, small, fast-moving *Bdellovibrio* cells search for Gram-negative prey bacteria and attach to the prey’s outer surface. To invade the prey cell, *Bdellovibrio* use lytic enzymes to open a small pore in the prey outer membrane and cell wall (5), then they move into the periplasmic space. During this process, peptidoglycan-modifying enzymes secreted by *Bdellovibrio* remodel the prey cell wall (6), reforming the prey cell into a rounded bdelloplast and eventually resealing the pore after *Bdellovibrio* are established in the periplasm. The remodeled prey peptidoglycan prevents invasion by other attack-phase *Bdellovibrio* and ensures that only the resident *Bdellovibrio* can use prey cell contents as nutrients. In the context of the experimental evolution scheme used here, this creates a competition favoring individuals that are more successful at finding, invading, and digesting prey to produce progeny.

Once *Bdellovibrio* have successfully invaded prey, they initiate the growth phase of the life cycle, secreting a suite of lytic enzymes into the prey cytoplasm to digest macromolecules for use as nutrients. *Bdellovibrio* species are distinguished from their nonpredatory relatives by the extensive number of lytic enzymes encoded in their genomes (7). During this stage, *Bdellovibrio* grows as a long, filamentous cell; when the prey cytoplasm is exhausted, this cell septates into multiple progeny (8, 9). To complete the life cycle, progeny exit the prey cell using lytic enzymes that target prey peptidoglycan (10). In the context of the experimental evolution scheme used here, reduced availability of susceptible Gram-negative prey bacteria may favor individuals with more effective mechanisms to survive periods of nutrient limitation, such as reported in (11).

As an experimental strategy, maintaining microbial populations over many generations under selective pressures associated with defined laboratory conditions has provided important insights into the evolution of many microbes, including bacteria (12, 13), yeast (14), and bacteriophage (15). Considering predatory bacteria, most experimental evolution studies have focused on *Myxococcus*, which is a facultative “wolfpack” predator (16, 17). Two connected studies applied an experimental evolution approach to examine coevolution between B. bacteriovorus and Pseudomonas fluorescens (18, 19); however, genome sequencing has not yet been leveraged to reconstruct the evolutionary trajectories of *Bdellovibrio* populations during experimental evolution. Here, we established six replicate populations of *Bdellovibrio* sp. NC01 and used serial passaging of attack-phase cells to investigate *Bdellovibrio* evolution during long-term coculture with a Pseudomonas strain as prey. Our experimental design did not allow Pseudomonas to evolve in response to predation, enabling us to focus on how *Bdellovibrio* responds to a single, unchanging prey strain.

## RESULTS

### *Bdellovibrio* sp. NC01 is not a highly efficient predator of Pseudomonas sp. NC02.

We previously reported predation efficiency data for *Bdellovibrio* sp. NC01 using Escherichia coli ML35 as prey (3); however, NC01 is unlikely to encounter E. coli in its native soil habitat. Here, we tested the ability of NC01 to kill Gram-negative soil bacteria by using Pseudomonas sp. NC02 as prey. Not only was this strain of Pseudomonas originally isolated from soil (20), but it was also used as prey during the initial stages of *Bdellovibrio* sp. NC01 isolation, as detailed in Materials and Methods. Determining NC01 predation efficiency on Pseudomonas sp. NC02 expands our data on variation in *Bdellovibrio* predatory phenotypes. To reduce confusion regarding strain names, we will here refer to Pseudomonas sp. NC02 as simply “Pseudomonas.”

**FIG 1:**
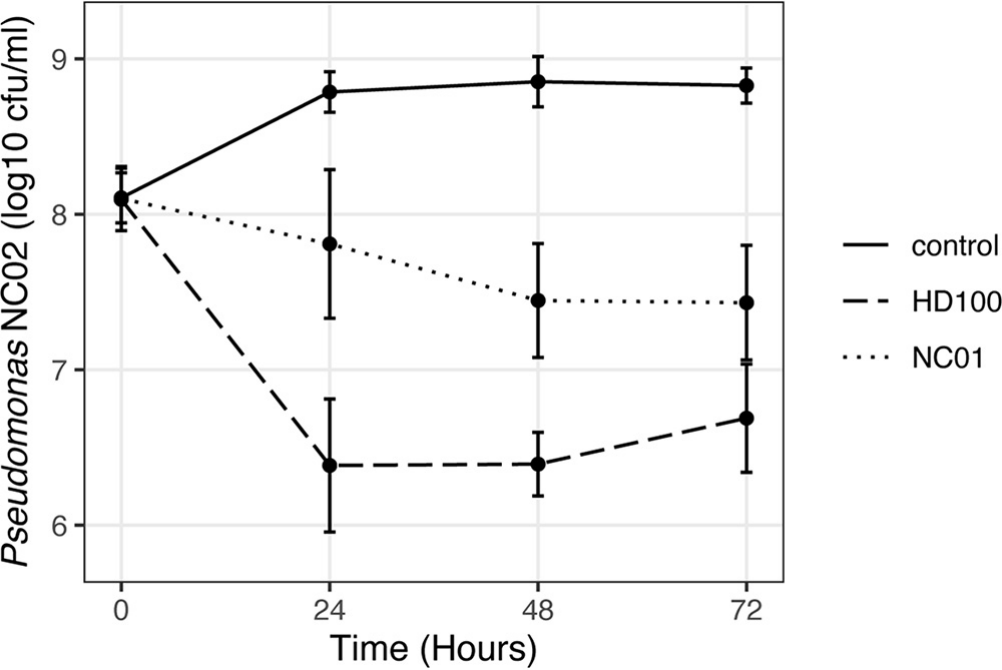
Predation efficiency of *Bdellovibrio* sp. NC01 and B. bacteriovorus HD100 on Pseudomonas. We quantified viable Pseudomonas sp. NC02 over 72 h in HM buffer (control), in HM buffer combined with *Bdellovibrio* sp. NC01 (NC01), and in HM buffer combined with B. bacteriovorus HD100 (HD100). The resulting CFU/mL data were log transformed and plotted using ggplot in RStudio. Each data point shows the mean and standard deviation for at least five replicates.

We quantified the ability of *Bdellovibrio* sp. NC01 to kill Pseudomonas in HM buffer over 72 h of coculture (Fig. 1). For comparison, we also tested the type strain B. bacteriovorus HD100. Both NC01 and HD100 caused reductions of the viable Pseudomonas population compared to the control. The maximum reduction in Pseudomonas CFU/mL was approximately 1.5 log units for NC01 at 48 and 72 h and 2.5 log units for HD100 at 24 and 48 h. Overall, the effects of both NC01 and HD100 on Pseudomonas prey populations are smaller than their previously observed effects on E. coli ML35 prey populations (3). Given that *Bdellovibrio* are capable of reducing some prey populations by up to 8 log units (21), these predation efficiency data suggest that neither of the *Bdellovibrio* strains tested here are highly efficient predators of this particular Pseudomonas strain.

### Experimental evolution provides a means to test how *Bdellovibrio* sp. NC01 responds to long-term coculture with Pseudomonas.

**FIG 2:**
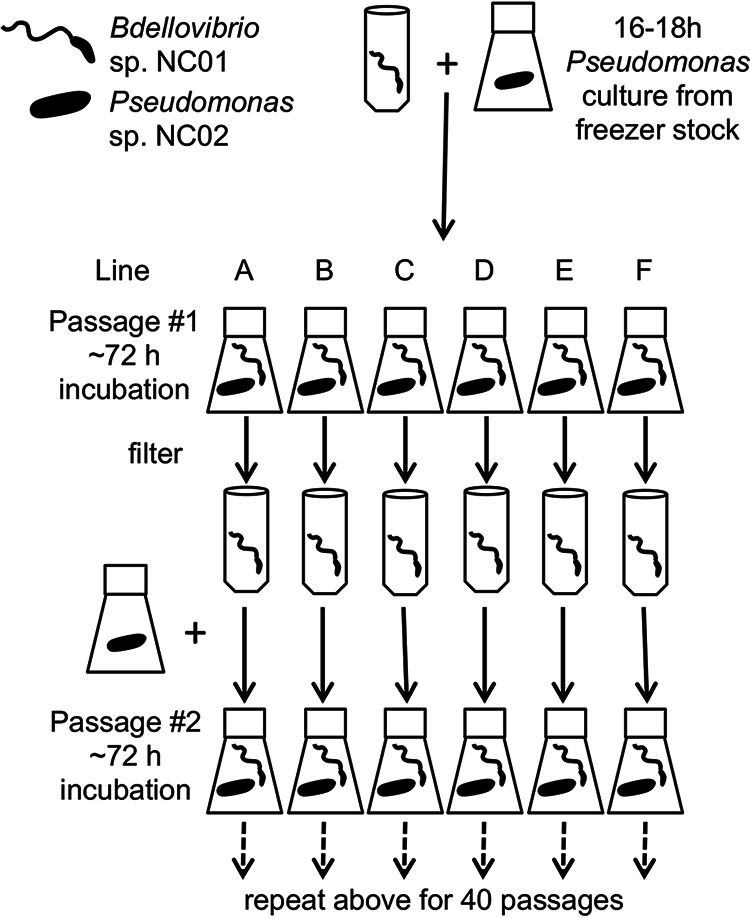
Experimental design for serial passaging of *Bdellovibrio* populations with a single Pseudomonas strain as prey.

We designed an experimental evolution scheme to investigate how *Bdellovibrio* evolves in response to long-term coculture with Pseudomonas (Fig. 2). We established six replicate lines (A to F) by combining aliquots of a single population of *Bdellovibrio* sp. NC01 with Pseudomonas grown from a freezer stock. After 72 h of incubation, during which NC01 invaded and digested Pseudomonas cells to produce progeny, we filtered the lysates to harvest *Bdellovibrio* attack-phase cells, then combined some of those cells with Pseudomonas freshly grown from the freezer stock. This experimental design allows the *Bdellovibrio* populations to evolve, but it does not allow the Pseudomonas population to evolve. Because each line is maintained separately from the others during passaging, the *Bdellovibrio* populations diverge from their NC01 ancestor following distinct evolutionary trajectories, enabling us to test how this *Bdellovibrio* strain responds to long-term coculture with a single, unchanging prey strain.

In total, we serially passaged each of the six lines 40 times, resulting in approximately 2,880 h of coculture for each line. Estimating the number of *Bdellovibrio* generations that occurred over the course of the experiment is complicated by multiple factors, such as variation in the number of progeny produced when *Bdellovibrio* invade and digest Pseudomonas cells. Here, we use passages rather than generations to track evolution of the *Bdellovibrio* populations. At regular time points over the course of the experiment, we froze aliquots of the filtered attack-phase *Bdellovibrio* cells when passaging the lines. The resulting freezer stocks provided a resource for genotypic and phenotypic analyses, enabling us to reconstruct the evolutionary trajectories of the six lines.

### Nonsynonymous substitutions were the most abundant type of new mutation detected in *Bdellovibrio* populations.

We generated short-read sequencing data from freezer stocks of the *Bdellovibrio* populations established at six time points and then used the software package *breseq* to analyze the reads and detect new mutations. We distinguished unique new mutations within each line by their position on the *Bdellovibrio* sp. NC01 genome and the changes they cause in the nucleotide sequence. If a mutation was detected within a line at a particular time point, not detected at a subsequent time point, then detected again at a later time point, we analyzed this as one new mutation for the line, assuming that the *Bdellovibrio* population experienced fluctuations in the mutation’s frequency rather than two independent occurrences of the same exact mutation. This is consistent with how *breseq* reports mutations. Our settings for *breseq* included a frequency cutoff of 0.05, therefore mutations present at <5% in the *Bdellovibrio* populations are not reported. This cutoff is recommended to reduce the likelihood of false positives (https://barricklab.org/twiki/pub/Lab/ToolsBacterialGenomeResequencing/documentation/methods.html).

**FIG 3:**
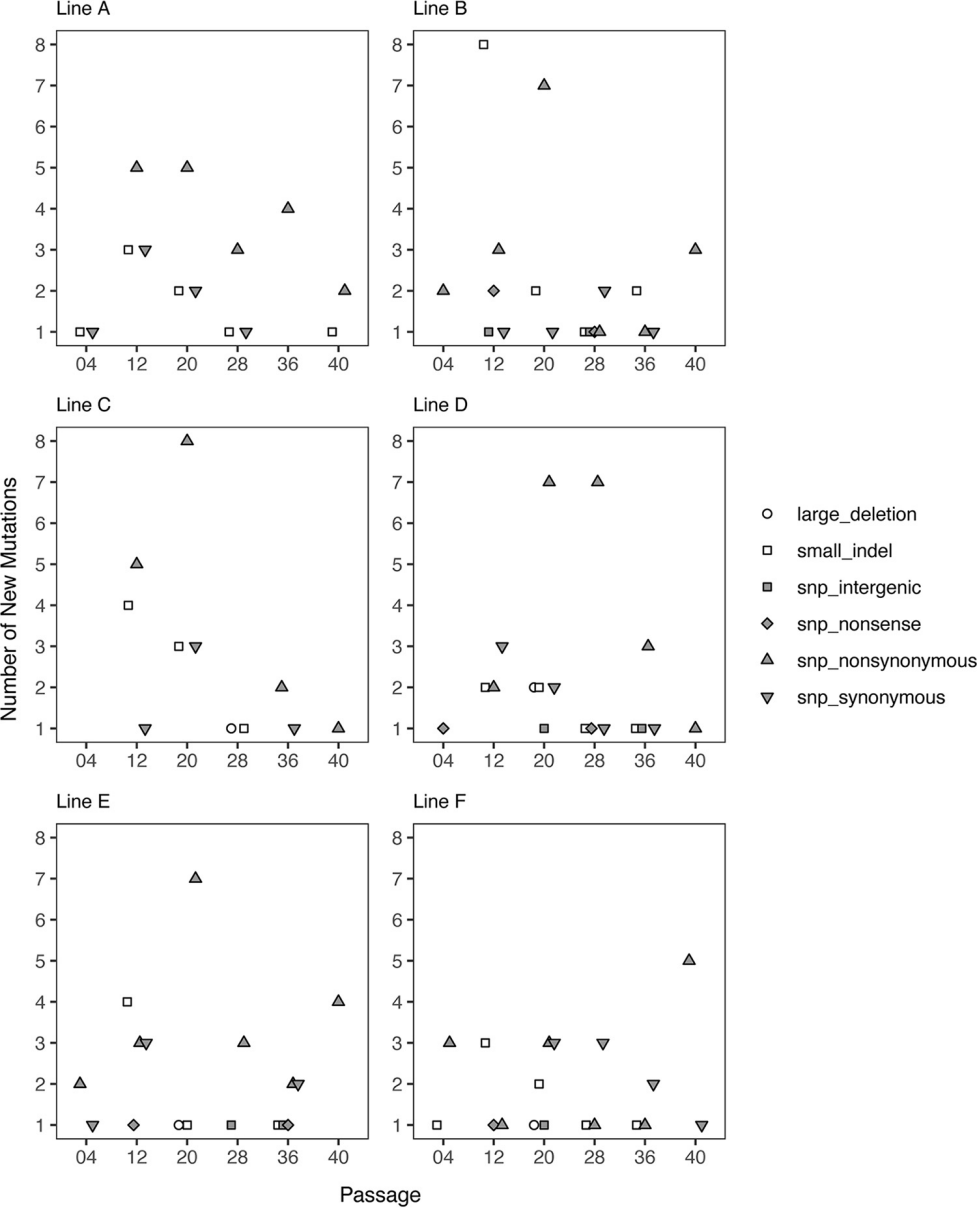
New mutations detected in *Bdellovibrio* populations during experimental evolution. Data points are plotted to show the number of new mutations of various types detected within each line at different time points over the course of the experiment. As indicated by the shape and color of the data points, mutation types are as follows: single-nucleotide substitutions causing an amino acid change in a protein-coding gene (snp_nonsynynomous), single-nucleotide substitutions that occur in a protein-coding gene but do not cause an amino acid change (snp_synonymous), single-nucleotide substitutions that introduce a premature stop codon in a protein-coding gene (snp_nonsense), single-nucleotide substitutions occurring in a region that is not predicted to encode a protein (snp_intergenic), small insertions or deletions totaling <15 bp (small_indel), and large deletions involving >50 bp (large_deletion).

On average, we detected six new mutations at each time point in each line (range 1 to 15, Fig. 3). Considering all six time points together, we detected a total of 34 new mutations in line A, 40 in line B, 30 in line C, 39 in line D, 38 in line E, and 34 in line F, which suggests that the *Bdellovibrio* populations experienced a similar mutation rate. In each line, the majority of new mutations were detected at only a single time point (Table S1), indicating maintenance of a low level of genetic diversity in the *Bdellovibrio* populations during long-term coculture with Pseudomonas. Given the PGAP (Prokaryotic Genome Annotation Pipeline) annotation of the ancestor *Bdellovibrio* sp. NC01, the most abundant type of new mutation detected in the populations was nonsynonymous single-nucleotide substitutions, which accounted for 41 to 56% of all new mutations detected in each line. The next most abundant types of new mutations were small (<15 bp) insertions or deletions (15 to 33% of all new mutations detected in each line) and synonymous single-nucleotide substitutions (13 to 26%). In addition, we detected single-nucleotide substitutions that cause premature stop codons, single-nucleotide substitutions in intergenic regions, and large (>50 bp) deletions; however, these mutation types were rarer in the *Bdellovibrio* populations, with only three or fewer new mutations of each type detected in each line over the course of the experiment.

### Most mutations that reached high frequency in evolved *Bdellovibrio* populations are predicted to alter protein sequences.

**FIG 4:**
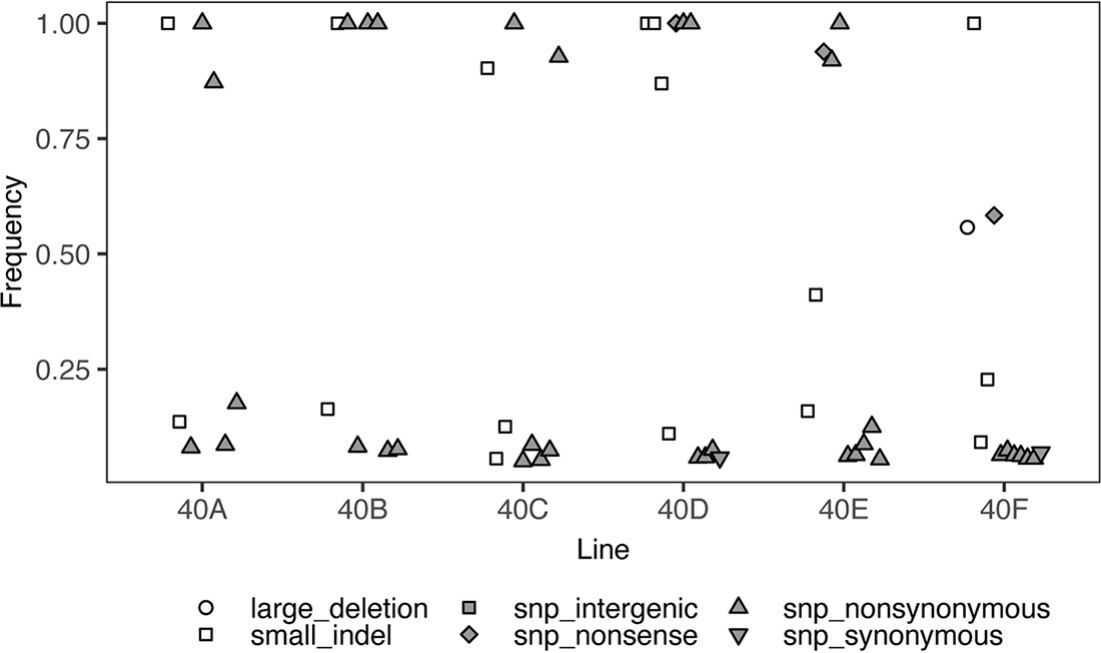
Frequency of mutations detected in the evolved *Bdellovibrio* populations. All mutations detected at ≥5% in the *Bdellovibrio* populations after 40 passages are plotted by frequency. Mutation types are indicated by the shape and color of the data points, following the definitions outlined in Fig. 3. Due to the polymorphism-frequency-cutoff setting of 0.05, *breseq* did not report mutations detected at <5%, and mutations detected at ≥95% were reported at 100%. To visually distinguish mutations with similar frequencies within each line, we dodged data points on the *x* axis in ggplot.

Because of the large *Bdellovibrio* population sizes in this experiment, a mutation with very small beneficial effect, no beneficial effect, or deleterious effect is likely to be lost by genetic drift or eliminated by selection before reaching detectable frequency (>5%) in a population, unless it is linked to a mutation with a substantial beneficial effect (22, 23). Mutations that reach a frequency of 5% in the *Bdellovibrio* populations are subject to competition; therefore, mutations with the largest selection coefficients, and any mutations linked to them within the same genetic background, are most likely to reach high frequencies in the populations. We examined all mutations that were present at a frequency of >5% in each line after 40 passages, which constituted the endpoint of the experiment (Fig. 4). We refer to the *Bdellovibrio* populations at this time point as the evolved *Bdellovibrio* populations. Almost all the mutations detected in the evolved *Bdellovibrio* populations were present at either low frequency (here, defined as <25%) or high frequency (here, defined as >75%). Only three mutations reached an intermediate frequency (25 to 75%): a small indel in line E, a large deletion in line F, and a single-nucleotide substitution that causes a premature stop codon in line F. The number of high-frequency mutations detected in the evolved *Bdellovibrio* populations ranged from one (line F) to six (line D). Overall, we detected 20 high-frequency mutations across all six lines at the endpoint of the experiment.

These high-frequency mutations comprised 11 nonsynonymous single-nucleotide substitutions, seven small indels, and two single-nucleotide substitutions that cause premature stop codons (Fig. 4 and Table 1). Both types of substitutions will alter the amino acid sequences of the affected genes’ protein products. Of the seven small indels, only two occurred in intergenic regions, where they will not have a direct impact on amino acid sequences, whereas five occurred within protein-coding genes. Four of these were single nucleotide indels, which will cause a frameshift during translation and therefore alter amino acid sequences. We examined the context of the single nucleotide indels and determined that none of them occur within a homopolymer longer than two nucleotides, which suggests that it is unlikely for polymerase slippage to correct the frameshifts. The other small indel that occurred within a protein-coding gene was a 6-nt deletion, which will alter the amino acid sequence but will not result in a frameshift. In total, we predict that 18 of the 20 high-frequency mutations detected in the evolved *Bdellovibrio* populations will result in changes to protein sequences.

**TABLE 1:** High-frequency (>75%) mutations detected in evolved *Bdellovibrio* populations^*a*^

Locus tag	PGAP annotation	Evolved line	Mutation position	Mutation category	Ancestral sequence	Evolved sequence	AA change (if applicable)
**DOE51_RS00275**	**Metallophosphoesterase**	**D**	**53,142**	**snp_nonsense**	**C**	**T**	Q156*
	**Metallophosphoesterase**	**B**	**53,967**	**snp_nonsyn**	**C**	**T**	**H431Y**
**DOE51_RS02140**	**TonB family protein**	**C**	**422,727**	**small_indel**	**A**	.	
	**TonB family protein**	**F**	**422,898**	**small_indel**	**G**	**+T**	
DOE51_RS04215	Hypothetical protein	D	866,847	small_indel	CGCCAA	.	
DOE51_RS05495/DOE51_RS05500	Intergenic	D	1,142,145	small_indel	T	+G	
DOE51_RS05845	Prepilin-type N-terminal cleavage/methylation domain-containing protein	B	1,216,726	snp_nonsyn	C	A	N176K
**DOE51_RS07450**	**Hypothetical protein**	**A**	**1,558,689**	**small_indel**	**G**	.	
	**Hypothetical protein**	**E**	**1,559,309**	**snp_nonsyn**	**T**	**A**	**H48L**
DOE51_RS07835	Nucleotide sugar dehydrogenase	D	1,638,828	snp_nonsyn	C	T	R375C
DOE51_RS08315/DOE51_RS08320	Intergenic	D	1,729,016	small_indel	G	TGAG + 7	
DOE51_RS08710	TIGR04552 family protein	B	1,817,535	small_indel	A	.	
DOE51_RS09440	KUP/HAK/KT family potassium transporter	C	1,958,870	snp_nonsyn	G	A	C548Y
DOE51_RS10010	Bifunctional alpha, alpha-trehalose-phosphate synthase (UDP-forming)/trehalose-phosphatase	E	2,085,242	snp_nonsense	G	A	Q464*
**DOE51_RS11010**	**Na/Pi cotransporter family protein**	**D**	**2,291,450**	**snp_nonsyn**	**G**	**T**	**S65I**
	**Na/Pi cotransporter family protein**	**E**	**2,291,537**	**snp_nonsyn**	**C**	**T**	**T94M**
	**Na/Pi cotransporter family protein**	**A**	**2,292,302**	**snp_nonsyn**	**T**	**C**	**V349A**
	**Na/Pi cotransporter family protein**	**B**	**2,292,484**	**snp_nonsyn**	**T**	**C**	**F410L**
DOE51_RS11275	Site-specific recombinase	A	2,351,293	snp_nonsyn	C	A	V345L
DOE51_RS18060	Isocitrate dehydrogenase	C	3,759,236	snp_nonsyn	G	A	G262S

a . indicates a deletion (for indels). Bold font indicates a gene with a high-frequency mutation in two or more evolved *Bdellovibrio* populations.

**FIG 5:**
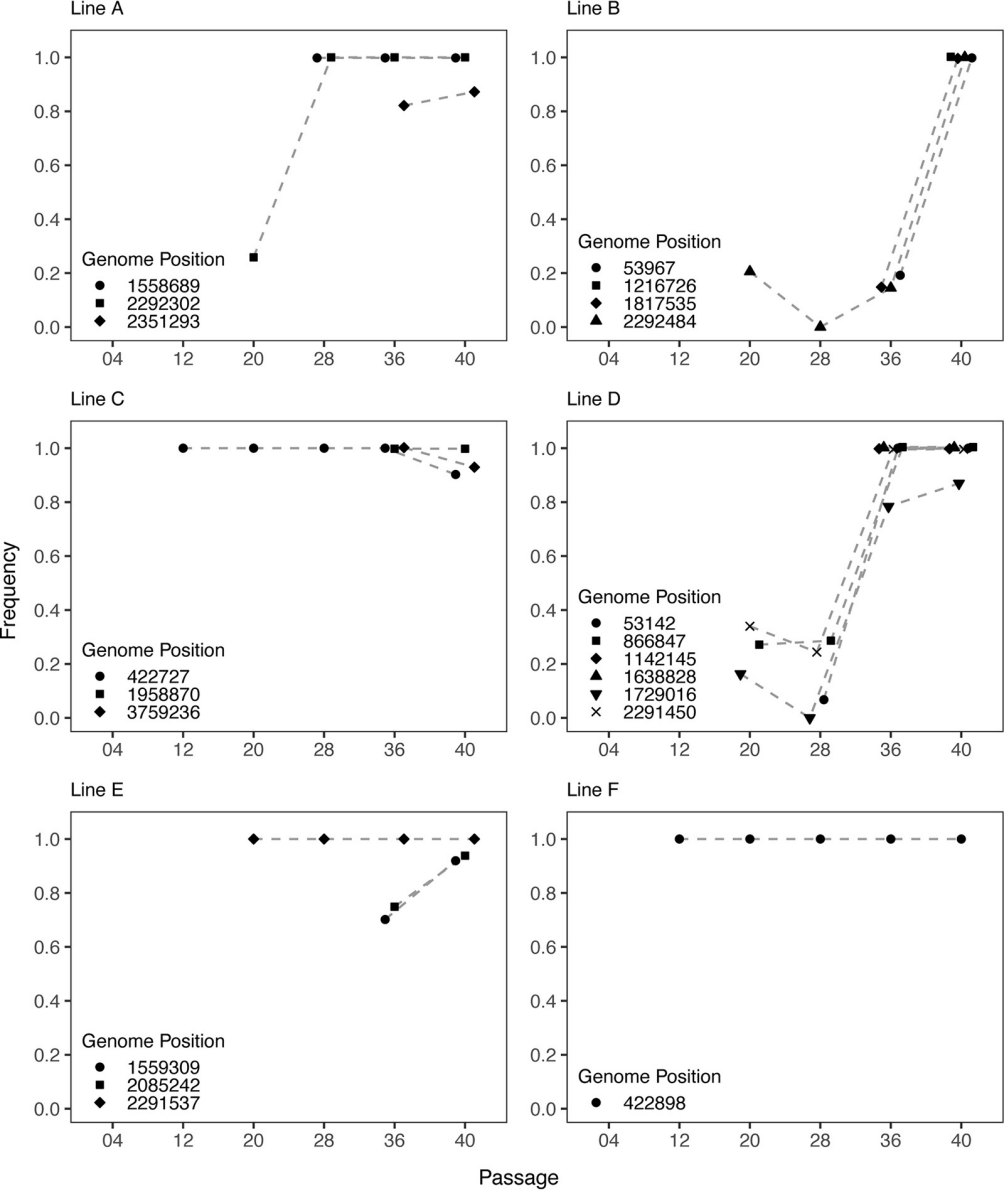
Trajectories of mutations that reached high frequency in the evolved *Bdellovibrio* populations. For each mutation that was detected at >75% in the *Bdellovibrio* populations after 40 passages, we plotted the frequency of the mutation over the course of the experiment, beginning with the time point at which the mutation was initially detected. Within each line, the genome position of the mutations is indicated by the shapes of the data points. Due to the polymorphism-frequency-cutoff setting of 0.05, *breseq* did not report mutations detected at <5%, which may explain the apparent disappearance of some mutations in lines B and D after their initial detection. Additionally, because of the cutoff setting, *breseq* reports mutations detected at ≥95% as present at 100%. To visually distinguish overlapping data points at particular time points, we dodged data points on the *y* axis by up to 0.004 in ggplot. The inset legend for each line does not obscure any data.

By analyzing short-read sequencing data from the six time points, we reconstructed the trajectories of the high-frequency mutations over the course of the experiment (Fig. 5). At the earliest time point, after only four passages, we did not detect any of the 20 mutations that eventually reached high frequency in the evolved *Bdellovibrio* populations. Just over half of these mutations (*n* = 11) were already at high frequency (>75%) when we detected them, and an additional quarter (*n* = 5) were initially detected at low or intermediate frequency, then reached high frequency before the next time point. Overall, 14 of the high-frequency mutations were detected at ≥95% in the evolved *Bdellovibrio* populations. The trajectories of these mutations, particularly their rapid increases in frequency, point to the role of positive selection under the conditions imposed during experimental evolution (24).

### Four genes acquired a high-frequency mutation in two or more of the evolved *Bdellovibrio* populations.

Based on the genome position of mutations and the PGAP annotation of the ancestor *Bdellovibrio* sp. NC01, we identified four protein-coding genes that acquired a high-frequency mutation in two or more of the *Bdellovibrio* populations by the endpoint of the experiment (Table 1). For each of these genes, the affected populations experienced unique high-frequency mutations; in other words, we never observed the exact same high-frequency mutation in the same gene in multiple *Bdellovibrio* populations. The mutations are all predicted to alter the amino acid sequences of the protein products encoded by the four genes. Although it is possible for mutations to occur in the same gene in two or more populations by chance during experimental evolution, it is very unlikely that such mutations would reach a frequency of >75% in each population in the absence of selection (24). The high frequencies of the mutations described here demonstrate that some of the *Bdellovibrio* populations experienced parallel evolution at four protein-coding genes in response to the selective pressures associated with long-term coculture with Pseudomonas. The products of the four genes are annotated in the NC01 ancestor as a sodium/phosphate cotransporter family protein, a metallophosphoesterase, a TonB family protein, and a hypothetical protein.

### Sodium/phosphate cotransporter family protein.

A gene predicted to encode a sodium/phosphate cotransporter family protein (locus tag: DOE51_RS11010) acquired a single high-frequency nonsynonymous substitution in four *Bdellovibrio* populations. In three of the populations (lines A, B, and D), we initially detected each nonsynonymous substitution at <50%, and the substitutions then reached ≥95% by the endpoint of the experiment (Fig. 5). In the fourth population (line E), the nonsynonymous substitution was already at ≥95% when initially detected, and it remained at this frequency for the duration of the experiment. The occurrence of mutations that impact the amino acid sequence of the sodium/phosphate cotransporter in four of the six *Bdellovibrio* populations and the observation that each of the mutations reached high frequency in the populations indicate that this *Bdellovibrio* gene experienced strong selective pressure during experimental evolution.

In the annotation of the ancestor *Bdellovibrio* sp. NC01, the sodium/phosphate cotransporter family protein (GenBank protein id: WP_142696613) is assigned functions related to inorganic ion transport and metabolism (COG1283). We used TBLASTN to align the NC01 protein sequence against six-frame translations of GenBank nucleotide records classified as *Bdellovibrio*; this analysis identified full-length protein sequences with high similarity (≥63% aa identity) in all 10 complete *Bdellovibrio* genomes in the database, suggesting that this sodium/phosphate cotransporter family protein is conserved within the genus. Within the B. bacteriovorus HD100 genome, a gene with locus tag Bd2221 encodes a protein product (GenBank protein id: WP_011164654) with 89% aa identity to the NC01 protein sequence. In the supplemental material accompanying the original HD100 genome publication (7), Bd2221 is listed under “Permease mediated transport: Anion transport, phosphate.” Based on RNA-Seq data from (25), expression of Bd2221 is specific to the growth phase in HD100.

DeepTMHMM (26) identified the sodium/phosphate cotransporter family protein as a membrane protein with 11 transmembrane alpha-helix domains, and AlphaFold (27) also predicted multiple alpha-helices. Based on DeepTMHMM analysis, only the mutations in lines D and E occurred within transmembrane domains. These two mutations affect different alpha-helices; however, the protein structure predicted by AlphaFold shows them in close proximity, albeit lacking any direct bonds. The mutations in lines A and B occurred in a different region of the protein, and they do not appear in close proximity to each other in the structure predicted by AlphaFold.

### Metallophosphoesterase.

A gene predicted to encode a metallophosphoesterase (locus tag: DOE51_RS00275) acquired a nonsynonymous single-nucleotide substitution in line B and a single-nucleotide substitution that causes a premature stop codon in line D. Both substitutions were initially detected at <20%, and both then reached ≥95% at the next time point. In the annotation of the ancestor *Bdellovibrio* sp. NC01, the metallophosphoesterase (GenBank protein id: WP_142694621) is assigned functions related to hydrolase activity (GO term 0016787). Using TBLASTN analysis, we identified full-length protein sequences with high similarity in all 10 complete *Bdellovibrio* genomes, including Bd0054 within the B. bacteriovorus HD100 genome, which is expressed during the growth phase based on RNA-Seq data from (25). Whereas the NC01 protein sequence aligned to protein sequences of other intraperiplasmic *Bdellovibrio* with >67% aa identity, there was greater divergence between the NC01 protein sequence and protein sequences of epibiotic *Bdellovibrio* strains JSS and qaytius, as each strain’s sequence aligned with ~48% aa identity to that of NC01.

DeepTMHMM identified the metallophosphoesterase as a membrane protein with two transmembrane alpha-helix domains. AlphaFold also predicted alpha-helices at the same residues. The mutations in lines B and D did not occur within these transmembrane domains, and they are not in close proximity based on the structure predicted by AlphaFold.

### TonB family protein.

A gene predicted to encode a TonB family protein (locus tag: DOE51_RS02140) acquired a high-frequency single-nucleotide indel between passage 4 and passage 12 in two *Bdellovibrio* populations (lines C and F). In the ancestor NC01, the TonB family protein sequence is 324 amino acids long; however, the indel in line F causes a frameshift after 221 amino acids, whereas the indel in line C causes a frameshift after 278 amino acids. When initially detected, both indels were already at ≥95% in the populations. The indel in line F remained at this frequency for the duration of the experiment, whereas the indel in line C remained at this frequency over the next three time points but then decreased to 90% at the endpoint of the experiment.

In the annotation of the ancestor *Bdellovibrio* sp. NC01, the TonB family protein (GenBank protein id: WP_142694953) is assigned functions related to transport, including “secondary active transmembrane transporter activity” (GO term 0015291). Using TBLASTN analysis, we identified highly similar genes in each of the 10 complete *Bdellovibrio* genomes, including the TonB family protein Bd0396 in B. bacteriovorus HD100. In the supplemental material accompanying the original HD100 genome publication (7), Bd0396 is listed under “Permease mediated transport: iron.” Based on RNA-Seq data from (25), expression of Bd0396 is specific to the attack phase in HD100. The NC01 protein sequence aligned by TBLASTN to genes annotated as functional in every complete *Bdellovibrio* genome in the database; however, the alignment to B. bacteriovorus 109J was not full-length, spanning 294 of 324 aa. This is unusual, given that 109J is closely related to other *Bdellovibrio* strains, such as HD100 and Tiberius, whose homologous genes aligned along their entire length. In contrast to the frameshift mutations detected in lines C and F, which occurred in the latter half of the sequence, the 109J protein seems to have a shortened N-terminal region.

DeepTMHMM predicted a single transmembrane alpha-helix within the first 30 amino acids of the NC01 TonB family protein, and this alpha-helix is also predicted by AlphaFold. The mutations in lines C and F did not occur in this domain. To determine if data on TonB expression or protein structure can inform predictions about the functional effects of the frameshift mutations, we aligned the TonB family protein from NC01 against Bd2860 (WP_144313868) from HD100, which is annotated as the energy transducer TonB. The NC01 protein aligned to only 27% of Bd2860 at only 21% identity, which is a low-scoring alignment with a relatively large E value (0.002). These alignment results show that, despite the TonB family C-terminal domain identified by Hidden Markov Models during genome annotation, this NC01 protein is highly divergent from *Bdellovibrio* TonB.

### Hypothetical protein.

A gene predicted to encode a hypothetical protein of 375 amino acids (locus tag: DOE51_RS07450) acquired a single-nucleotide deletion in line A and a single-nucleotide substitution in line E. We initially detected the deletion in line A at ≥95%, and it remained at this frequency until the endpoint of the experiment, whereas the substitution in line E first appeared at 70% at passage 36 then increased in frequency to 92% by the endpoint of the experiment. This hypothetical protein (GenBank protein id: WP_142695917) has no functional annotation information associated with it. DeepTMHMM did not predict any transmembrane domains. TBLASTN analysis identified full-length protein sequences with at least 30% amino acid identity in all 10 complete *Bdellovibrio* genomes. As with the metallophosphoesterase, we noted higher similarity (>73% aa id) between the NC01 protein sequence and those of other intraperiplasmic *Bdellovibrio*, including Bd1601 within the B. bacteriovorus HD100 genome, which is expressed during the growth phase based on RNA-Seq data from (25). The NC01 protein sequence aligned to protein sequences of epibiotic *Bdellovibrio* strains JSS and qaytius with lower similarity (31-34% aa id).

Overall, the evidence for parallel evolution is strongest for the sodium/phosphate cotransporter, which acquired a high-frequency mutation predicted to alter the protein sequence in four *Bdellovibrio* populations. For the other three protein-coding genes, the occurrence of mutations that reached and then maintained high frequency in two different *Bdellovibrio* populations suggests that these genes were also subject to strong positive selection during experimental evolution.

### Evolved *Bdellovibrio* populations do not differ significantly from ancestor in tests of prey range and predation efficiency.

To determine if long-term coculture with Pseudomonas resulted in measurable changes to predatory phenotypes, we performed tests of prey range and predation efficiency. For prey range, we tested the ability of each evolved *Bdellovibrio* population to form plaques on lawns of eight different Gram-negative bacteria, which were previously used to assess the prey range of the ancestor *Bdellovibrio* sp. NC01. All the evolved *Bdellovibrio* populations formed plaques on the same five Gram-negative strains as the ancestor (Table 2). None of the evolved *Bdellovibrio* populations acquired the ability to form plaques on any of the three Gram-negative strains that were not killed by the ancestor.

**TABLE 2:** Prey range of *Bdellovibrio* sp. NC01 ancestor and evolved *Bdellovibrio* populations

Classification	Strain ID	Environment	Plaque formation^*a*^						
			NC01	40A	40B	40C	40D	40E	40F
Acinetobacter	0036	Freshwater	No	No	No	No	No	No	No
*Aeromonas*	0031	Freshwater	Yes	Yes	Yes	Yes	Yes	Yes	Yes
Enterobacteriaceae	0032	Freshwater	Yes	Yes	Yes	Yes	Yes	Yes	Yes
*Raoultella*	0037	Freshwater	No	No	No	No	No	No	No
Pseudomonas	NC02^*b*^	Soil	Yes	Yes	Yes	Yes	Yes	Yes	Yes
*Serratia*	0043	Soil	Yes	Yes	Yes	Yes	Yes	Yes	Yes
Escherichia	0057		No	No	No	No	No	No	No
Escherichia	ML35		Yes	Yes	Yes	Yes	Yes	Yes	Yes

a Plaque formation data for evolved Bdellovibrio populations based on one replicate.

b *Pseudomonas* sp. NC02 was previously referred to as strain 0042.

**FIG 6:**
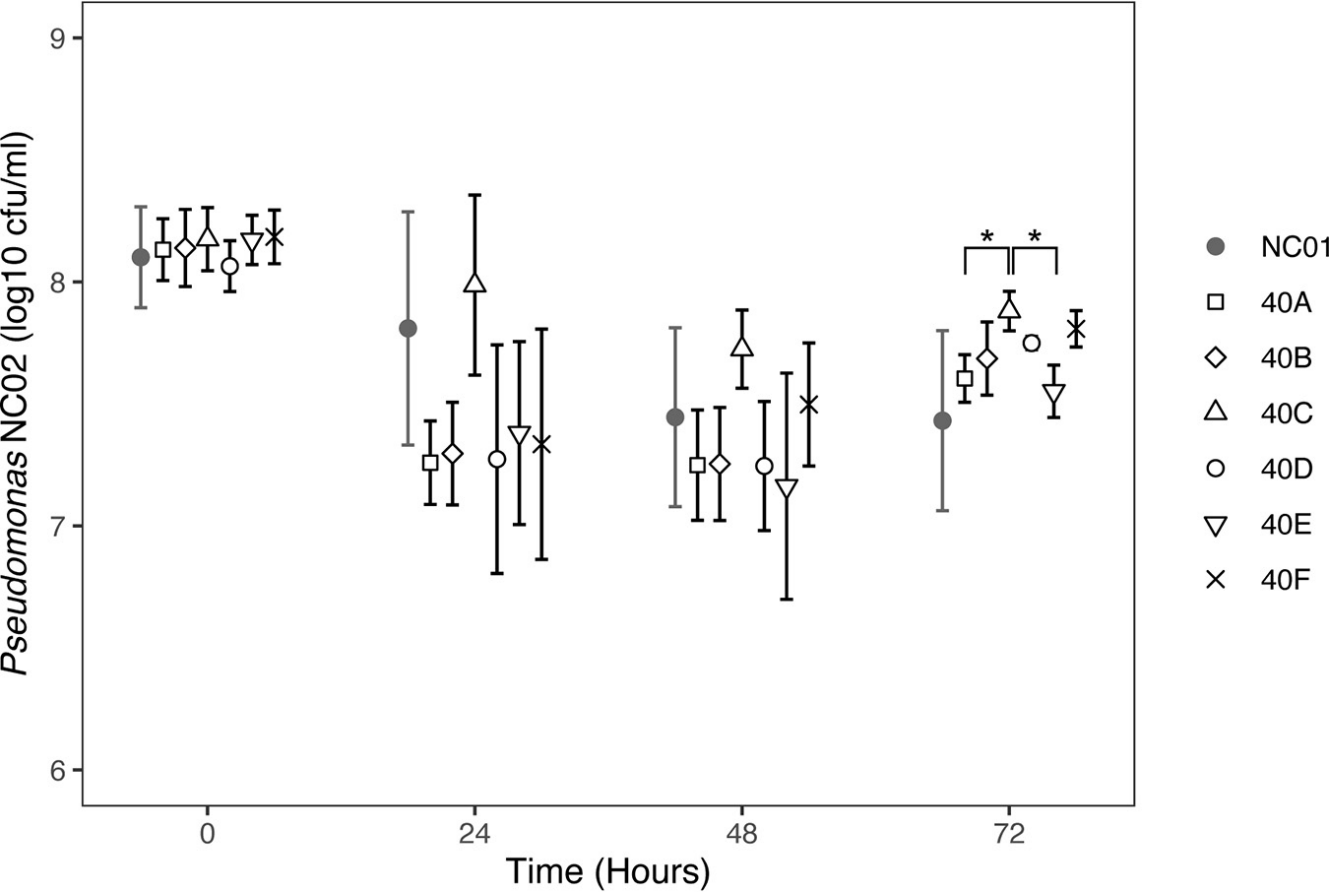
Predation efficiency of evolved *Bdellovibrio* populations on Pseudomonas. After 40 passages, we quantified the evolved *Bdellovibrio* populations’ ability to kill Pseudomonas using the same assay as described for Fig. 1. The resulting CFU/mL data were log transformed and plotted using ggplot in RStudio. The mean and standard deviation of four replicates are shown for each evolved population at each time point. Predation efficiency data from Fig. 1 for the ancestor *Bdellovibrio* sp. NC01 are plotted in gray for comparison. One-way ANOVA followed by a *post hoc* Tukey’s HSD test identified statistically significant differences (*P* < 0.01, indicated by asterisk) between 40C and 40A and between 40C and 40E. No other statistically significant differences in predation efficiency were identified.

For predation efficiency, we quantified the ability of each evolved population to kill Pseudomonas in HM buffer over 72 h (Fig. 6). After 24 h, the average concentration of viable Pseudomonas was lower when cocultured with five of the six evolved *Bdellovibrio* populations (A, B, D, E, and F) than when cocultured with the ancestor. Four of these populations (A, B, D, and E) maintained a lower average concentration of viable Pseudomonas compared to the ancestor after 48 h of coculture. By contrast, coculture with evolved population C yielded a higher average concentration of viable Pseudomonas compared to the ancestor at 24 and 48 h. After 72 h, the average concentration of viable Pseudomonas was higher for all six evolved *Bdellovibrio* populations compared to the ancestor.

To determine whether any of these observed differences were statistically significant, we performed 36 pairwise t-tests comparing the log-transformed Pseudomonas CFU/mL data for each evolved population to that of the ancestor at 24, 48, and 72 h in the predation efficiency assay. None of these t-tests identified statistically significant differences (*P* > 0.01 for all t-tests). To determine whether any of the evolved *Bdellovibrio* populations differed significantly from the others, we used one way ANOVA to compare log-transformed Pseudomonas CFU/mL data for all the evolved populations. We analyzed each time point in the predation efficiency assay independently, and, for each time point, we confirmed that the log-transformed data met the assumption of normality using the Shapiro-Wilk test (*P* > 0.01) and the assumption of equal variances using Levene’s test (*P* > 0.01). One way ANOVA identified statistically significant difference (*P* < 0.01) among the six evolved populations only at 72 h. A *post hoc* Tukey’s HSD test analyzing all possible pairwise comparisons among the six evolved populations at this time point demonstrated that predation efficiency of 40C differed significantly (*P* < 0.01) from that of both 40A and 40E.

In addition to testing predation efficiency by quantifying the viable prey population during coculture, we also quantified *Bdellovibrio* at the start (0 h) and end (72 h) of the assays (Fig. S1). After 3 days of coculture with Pseudomonas, concentrations of *Bdellovibrio* increased by 0.5–0.8 log units, but we did not detect any statistically significant differences among the ancestor and evolved *Bdellovibrio* populations.

Overall, these data indicate that long-term coculture with Pseudomonas did not result in significant changes in the evolved *Bdellovibrio* populations’ ability to kill Pseudomonas. This suggests that the high-frequency mutations detected in the evolved populations do not produce substantial improvements in predation efficiency on Pseudomonas compared to the ancestral phenotype.

### Evolved *Bdellovibrio* populations are better at surviving starvation than ancestor.

**FIG 7:**
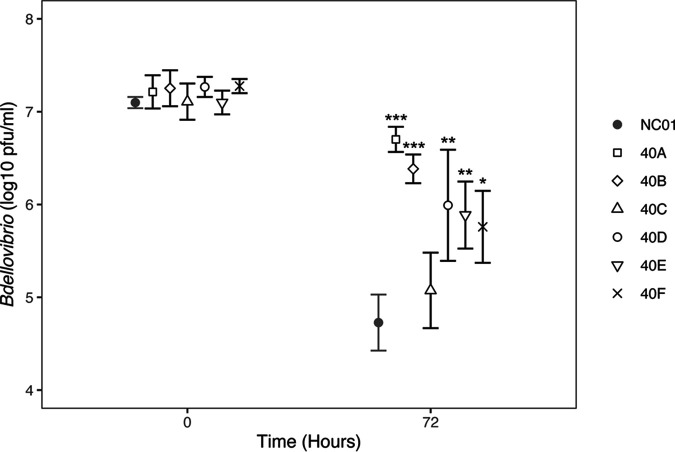
Decrease of viable *Bdellovibrio* after 72 h in the absence of prey. To test ancestral and evolved *Bdellovibrio*’s ability to survive starvation, we quantified *Bdellovibrio* by plaque counts at the start (0 h) and end (72 h) of incubation in HM buffer without prey. The resulting PFU/mL data were log transformed and plotted using ggplot in RStudio. One-way ANOVA followed by a *post hoc* Tukey’s HSD test identified statistically significant differences between the ancestor *Bdellovibrio* sp. NC01 and five of the evolved *Bdellovibrio* populations (*P* < 0.001 indicated by ***, *P* < 0.01 indicated by **, and *P* < 0.05 indicated by *). The *post hoc* test also identified statistically significant differences between 40C and 40A (*P* < 0.001), 40C and 40B (*P* < 0.01), and 40C and 40D (*P* < 0.05).

We tested the survival of the ancestor *Bdellovibrio* sp. NC01 and the six evolved *Bdellovibrio* populations in HM buffer alone, which constitutes starvation conditions. Starting concentrations of *Bdellovibrio* ranged from 8.2 × 10^6^–2.8 × 10^7^ PFU/mL. After 72 h of incubation in HM buffer without prey, the ancestor NC01 experienced an average decrease in the viable population of 2.4 log units, whereas each of the six evolved *Bdellovibrio* populations experienced a smaller decrease (Fig. 7). One-way ANOVA and *post hoc* analysis identified statistically significant differences between the ancestor and five evolved populations (A, B, D, E, and F), which each decreased by only 0.5–1.5 log units on average. For the sixth population (C), the average concentration of viable *Bdellovibrio* remaining after 72 h was greater than that of the ancestor, but the difference was not statistically significant (*p* > 0.05). These data show that long-term coculture of *Bdellovibrio* sp. NC01 with Pseudomonas, which it cannot efficiently kill, resulted in adaptation to nutrient-limited conditions and evolution of an enhanced ability to survive starvation.

## DISCUSSION

Experimental evolution offers a powerful tool to understand the predatory lifestyle of *Bdellovibrio* and define mechanisms underlying variation in predatory phenotypes. We observed that *Bdellovibrio* sp. NC01 and B. bacteriovorus HD100 vary in their ability to kill the Gram-negative soil bacterium Pseudomonas sp. NC02, with HD100 slightly more effective at killing Pseudomonas than NC01. Neither *Bdellovibrio* strain reduced the Pseudomonas prey population to the same extent as previously observed when using E. coli ML35 as prey (3). Interactions between predatory bacteria and prey bacteria are affected by predator-specific mechanisms and prey-specific mechanisms that combine to shape the outcome. Experimental evolution provides a means to identify these mechanisms as bacteria adapt to different selective conditions related to predation. Here, we applied an experimental evolution approach to test how *Bdellovibrio* sp. NC01 responds to long-term coculture with Pseudomonas and determine which *Bdellovibrio* genes are involved in adaptation to coculture conditions. After establishing six replicate lines with NC01 as the ancestor, we cocultured the *Bdellovibrio* populations with only Pseudomonas as prey for 40 passages, totaling approximately 2,880 h.

By growing Pseudomonas from freezer stock for each passage and transferring only attack-phase *Bdellovibrio* cells, the experimental evolution scheme used here focused solely on *Bdellovibrio* evolution, rather than coevolution between predatory bacteria and prey. The presence of a single, unchanging prey strain, which has reduced susceptibility to NC01 predation compared to other prey such as E. coli ML35, imposed selective pressures on the *Bdellovibrio* populations. For example, because *Bdellovibrio* compete for prey by rendering an invaded prey cell inaccessible to other attack-phase cells, selection should favor *Bdellovibrio* mutants with improved ability to invade and kill Pseudomonas and thereby produce progeny. However, it is also possible for selection to favor *Bdellovibrio* mutants that kill Pseudomonas at the same rate but produce more progeny from each invasion event. By contrast, given the poor predation efficiency of NC01 against Pseudomonas, selection may favor a different evolutionary strategy that improves survival during nutrient-limited conditions. The multiple selective pressures imposed by our experimental evolution design provide a powerful means to test the overall response of *Bdellovibrio* sp. NC01 to long-term coculture with Pseudomonas sp. NC02.

Using sequencing data from six time points over the course of the experiment, we detected an average of 36 new mutations in total for each *Bdellovibrio* population. Because of the large *Bdellovibrio* population sizes maintained during experimental evolution, each line will experience many mutations during each passage; however, the majority of these mutations will never reach a frequency greater than the 0.05 cutoff applied when analyzing sequencing data with *breseq* (22). Mutations reported here are either mutations with substantial beneficial effect or mutations that occurred within the same genome as a mutation with substantial beneficial effect, referred to as “hitchhiking” mutations. We detected mutations as early as passage 4, which was the first time point analyzed, emphasizing the rapidity of *Bdellovibrio*’s response to coculture with Pseudomonas. Some bacteria evolve hypermutability phenotypes during experimental evolution (23); however, the similar numbers of total detected mutations and mutations detected at each time point among the *Bdellovibrio* populations suggest that all six populations experienced a similar, stable mutation rate throughout the experiment.

Overall, nonsynonymous single-nucleotide substitutions were the most abundant type of new mutation detected in the *Bdellovibrio* populations, followed by small indels and synonymous single-nucleotide substitutions. Nonsynonymous mutations and small indels occurring within protein-coding genes will alter amino acid sequences, and the evolved proteins are subject to selection during experimental evolution. Synonymous substitutions do not directly affect the amino acid sequence of proteins, but these mutations can impact the structure and stability of mRNA transcripts (28), therefore synonymous substitutions may be subject to selection during experimental evolution. Some of the new mutations reported here may be hitchhiking mutations, which are not directly under selection themselves, but are linked on the same genome to a mutation or mutations that are under selection. The trajectories of mutations detected in the *Bdellovibrio* populations are shaped by their effects and the strength of selection imposed by long-term coculture with Pseudomonas. Even mutations that have a beneficial effect and reach a detectable frequency in a *Bdellovibrio* population may eventually disappear due to competition from other mutations that confer larger benefits. Over multiple passages, the mutations with the largest benefits, as well as any mutations hitchhiking with them, will persist and reach high frequencies in the *Bdellovibrio* populations.

After 40 passages, we detected 20 mutations at >75% in the evolved *Bdellovibrio* populations. With the exception of two small indels occurring in intergenic regions, all of these mutations directly affected amino acid sequences of proteins. We reconstructed the trajectories of these mutations using sequencing data from the six time points and determined that 16 of the 20 mutations were either already at high frequency when first detected or reached high frequency by the next time point. Such rapid increases in frequency within the *Bdellovibrio* populations suggest that these mutations were under strong positive selection during coculture with a single, unchanging Pseudomonas strain.

Half of the high-frequency mutations detected in the evolved *Bdellovibrio* populations occurred in four protein-coding genes. Each of these genes acquired a high-frequency mutation in two or more *Bdellovibrio* populations, indicating parallel evolution in response to long-term coculture with Pseudomonas. We never observed the exact same mutation in multiple *Bdellovibrio* populations; however, each mutation is predicted to alter the amino acid sequence of the gene’s protein product, which implicates the functions of the four proteins in *Bdellovibrio* adaptation during experimental evolution. These instances of parallel evolution demonstrate that, given the starting genotype of the ancestor *Bdellovibrio* sp. NC01, particular solutions are more likely than others as the populations adapt to selective conditions imposed by long-term coculture with a single, unchanging Pseudomonas strain.

For two of the four protein-coding genes, functional annotation information included evidence associated with transport. One of these genes is predicted to encode a sodium/phosphate cotransporter family protein, which experienced an amino acid replacement due to a high-frequency single-nucleotide substitution in four of the six evolved *Bdellovibrio* populations. This suggests that the sodium/phosphate cotransporter was under strong selection during long-term coculture with Pseudomonas. Based on BLAST analysis, the gene is homologous to Bd2221 in B. bacteriovorus HD100. There is very little information in the literature about this gene; however, Wurtzel and colleagues reported an amino acid replacement in the sodium/phosphate cotransporter in a host-independent isolate of B. bacteriovorus 109J (29). The amino acid replacement was one of 19 mutations detected in the host-independent isolate, which was able to grow in the absence of prey bacteria when supplied with adequate nutrients.

The other gene with functional annotation evidence associated with transport is predicted to encode a TonB family protein, which experienced a single-nucleotide indel causing a frameshift in two of the evolved *Bdellovibrio* populations. Based on BLAST analysis, the gene is homologous to Bd0396 in B. bacteriovorus HD100. Duncan and colleagues reported that a knockout mutation of Bd0396 in a B. bacteriovorus 109J background improved fitness in screens involving coculture with E. coli or V. cholerae, both planktonic and in biofilms (30). The knockout was constructed via transposon insertion in a host-independent isolate of 109J; therefore, it is unclear whether Bd0396 knockout was a compensatory mutation related to the mutations required for host independence or if Bd0396 knockout would improve fitness in the wild-type 109J background. Here, our data indicate that frameshift mutations in this TonB family protein in *Bdellovibrio* sp. NC01 were under strong positive selection as the *Bdellovibrio* populations adapted to long-term coculture with Pseudomonas.

Of the remaining two protein-coding genes that acquired a high-frequency mutation in multiple evolved *Bdellovibrio* populations, one of the genes is predicted to encode a metallophosphoesterase, which is homologous to Bd0054 in B. bacteriovorus HD100 based on BLAST analysis. Functional annotation of the NC01 genome associated the protein product with hydrolase activity. Lytic enzymes play key roles at multiple stages in the *Bdellovibrio* life cycle; however, one of the high-frequency mutations detected here is a single-nucleotide substitution that causes a premature stop codon, which suggests that loss of function may be adaptive under these conditions. The fourth gene is annotated in the NC01 genome as a hypothetical protein and lacks functional annotation information, preventing any hypotheses about its potential role in *Bdellovibrio* adaptation to long-term coculture with Pseudomonas. Genomes of *Bdellovibrio* have a substantial number of genes annotated as encoding hypothetical proteins, which complicates efforts to understand molecular mechanisms underlying predation.

Combined phenotype data for the evolved *Bdellovibrio* populations does not point to any significant alterations in prey range or predation efficiency as a result of long-term coculture with Pseudomonas. Prey range assays showed no differences between the NC01 ancestor and the evolved *Bdellovibrio* populations; however, prey range is scored as a binary based on plaque formation and may not change over the relatively short timescale examined here. Predation efficiency assays provide a quantitative measure of predatory ability that encompasses the extent and rate of killing. Compared to the NC01 ancestor, some of the evolved *Bdellovibrio* populations caused slightly larger reductions in viable Pseudomonas during 48 h of coculture; however, these differences were not statistically significant. Instead of evolving improved predatory phenotypes, *Bdellovibrio* adapted to the nutrient-limited conditions imposed by the experimental evolution design and evolved an enhanced ability to survive starvation. Compared to the ancestor NC01, all six evolved *Bdellovibrio* populations maintained higher concentrations of viable cells after 72 h of incubation in HM buffer without prey. The evolution of the same phenotype in multiple *Bdellovibrio* populations is yet another instance of parallel evolution in response to long-term coculture with Pseudomonas.

Overall, our analysis of short-read sequencing data suggests that *Bdellovibrio* sp. NC01 rapidly adapted to long-term coculture with Pseudomonas, with some populations arriving at the same solution to selective pressures imposed by experimental evolution. It may be possible to direct *Bdellovibrio* evolution in the lab to enhance predation efficiency on different prey species; our results point to potential modifications to the experimental design to test directed evolution, such as shortening the incubation time for each passage and extending the time frame to increase the total hours in coculture. The power and feasibility of experimental evolution, combined with the ease of genome sequencing, provides a valuable tool for defining mechanisms that govern variation in predatory phenotypes and will contribute to a deeper understanding of the predatory lifestyle of *Bdellovibrio*.

## MATERIALS AND METHODS

### Genome and predatory phenotypes of the ancestor *Bdellovibrio* sp. NC01.

We previously isolated *Bdellovibrio* sp. NC01 from a soil sample, sequenced the genome, determined prey range against a panel of eight Gram-negative bacteria, and quantified predation efficiency on E. coli ML35 (3). Here, we quantified the ability of NC01 to kill Pseudomonas sp. NC02 following the predation efficiency assay protocol described in (3), which is based on (31). For comparison, we also quantified predation efficiency of B. bacteriovorus HD100 on this prey strain. Pseudomonas sp. NC02 was isolated from soil collected at a different sampling location than that of NC01. Regarding NC01’s prior exposure to this prey strain, Pseudomonas sp. NC02 was used as prey during isolation of NC01 for initial enrichment and plaque formation, but a different prey strain classified as *Serratia* was used for subsequent rounds of lysates and plaque formation to obtain a pure isolate of NC01 and establish the freezer stock. We will refer to Pseudomonas sp. NC02 throughout this text as Pseudomonas to avoid confusion regarding NC01 and NC02 strain names.

Briefly, to quantify predation efficiency, we grew *Bdellovibrio* from −80°C freezer stocks using Pseudomonas as prey. After 72 h of incubation at 28°C and 200 rpm, we harvested attack-phase *Bdellovibrio* cells by filtering the lysates once, centrifuging the filtrates, then resuspending pelleted *Bdellovibrio* cells in sterile HM buffer (25 mM HEPES adjusted to pH 7.4 and supplemented with 3 mM calcium chloride dihydrate and 2 mM magnesium chloride hexahydrate). For prey, we used sterile HM buffer to dilute Pseudomonas from an overnight culture to an OD600 of 0.150. We then combined 500 μL of *Bdellovibrio* cells and 12.5 mL of diluted Pseudomonas cells to obtain lysates with an average *Bdellovibrio* concentration of 3.5 × 10^7^ cells/mL (based on direct cell counts) and an average Pseudomonas concentration of 1.4 × 10^8^ CFU/mL (based on serial dilution and colony counts). This yielded a predator:prey ratio of approximately 1:4. As a control, we combined 500 μL of sterile HM buffer with 12.5 mL of diluted Pseudomonas cells. We incubated the lysates at 28°C and 200 rpm and quantified viable Pseudomonas using CFU/mL counts at 24, 48, and 72 h.

### Experimental evolution of *Bdellovibrio* sp. NC01.

To establish the ancestral population of *Bdellovibrio* sp. NC01 for experimental evolution, we combined 25 mL sterile HM buffer, 1.5 mL of an overnight culture of E. coli ML35 grown in tryptic soy broth (TSB) at 37°C and 200 rpm, and a small amount of a −80°C freezer stock of *Bdellovibrio* sp. NC01. We incubated this lysate at 28°C and 200 rpm for approximately 72 h, then passed the lysate through a 0.45 μm filter to separate attack-phase *Bdellovibrio* cells from E. coli prey cells. To concentrate *Bdellovibrio* cells, we centrifuged the filtrate at 8,635 g (8,500 rpm in a Thermo Scientific A27 rotor) for 10 min, then resuspended the resulting pellet in 1.5 mL sterile HM buffer, which yielded 3.9 × 10^8^ *Bdellovibrio* cells/mL based on direct cell counts using a Petroff-Hausser counting chamber. This resuspension constituted the ancestral population of *Bdellovibrio* sp. NC01 for experimental evolution.

We established six replicate lines (named A-F) by combining an aliquot of the *Bdellovibrio* resuspension with Pseudomonas cells from a single prey population (Fig. 2). This ensures that the lines are as identical as possible at the start of the experiment. To obtain the Pseudomonas prey population, we streaked a small amount of a −80°C freezer stock of Pseudomonas sp. NC02 on a tryptic soy agar (TSA) plate and incubated the plate at 30°C for <24 h. We used colonies from this plate to inoculate an overnight culture in 15 mL TSB, which we incubated at 28°C and 200 rpm. We combined 10 mL of the Pseudomonas overnight culture with 20 mL sterile HM buffer, centrifuged this mixture at 8,635 g (8,500 rpm in a Thermo Scientific A27 rotor) for 10 min, then resuspended the pelleted Pseudomonas cells in sterile HM buffer to obtain an OD600 of 0.407, which yielded 1.77 × 10^9^ CFU/mL (based on serial dilution and colony counts). To establish the six replicate lines, we combined 200 μL of the *Bdellovibrio* sp. NC01 ancestral population described above with 25 mL of the Pseudomonas cell resuspension. Based on our concentration estimates, this resulted in a predator:prey ratio of approximately 1:1000 for these lysates, which we considered the first passage of the experiment. We incubated the lysates at 28°C and 200 rpm for ~72 h.

To perform serial passaging of the lines, we harvested attack-phase *Bdellovibrio* cells from the lysates and transferred some of these cells to a fresh Pseudomonas prey population. We grew Pseudomonas from the −80°C freezer stock for each passage, following the procedure described above. Because of this experimental design, the prey population encountered by *Bdellovibrio* throughout the experiment did not evolve. We harvested attack-phase *Bdellovibrio* cells by filtering lysates and centrifuging filtrates as described above. We resuspended *Bdellovibrio* pellets in 10 mL sterile HM buffer, then transferred 100 μL of the resuspensions to new flasks with fresh Pseudomonas prey at average OD600 of 0.550. Transferring 1% of the evolving population is consistent with methodology used by other bacterial experimental evolution projects (18, 19, 22, 23). Starting prey concentration for each passage ranged from 6.4 × 10^8^ to 2.8 × 10^9^ Pseudomonas CFU/mL (based on serial dilution and colony counts). We performed a total of 40 passages for all six lines following this serial passaging procedure.

To capture time points for sequencing and phenotype testing, we made freezer stocks of the *Bdellovibrio* populations at every fourth passage, starting with passage 4. After passaging the lines, we combined 500 μL of the remaining *Bdellovibrio* resuspensions with 500 μL 50% glycerol in cryovials and stored the stocks at −80°C. These freezer stocks also enabled us to restart the experiment when necessary. Early in the experiment, one of the lines became contaminated, and we used passage 4 freezer stocks to restart serial passaging. We also paused the experiment at passages 18 and 36 and then restarted serial passaging from freezer stocks made at these passages.

### Genome sequencing and analysis of *Bdellovibrio* populations.

To identify mutations arising during experimental evolution, we generated short-read sequencing data from freezer stocks of passages 4, 12, 20, 28, 36, and 40 for all six lines. We made lysates by combining 20 mL sterile HM buffer, 1.5 mL of an overnight culture of Pseudomonas sp. NC02 grown in TSB at 28°C and 200 rpm, and a small amount (~10 μL) of −80°C *Bdellovibrio* freezer stock, then incubated lysates for ~72 h at 28°C and 200 rpm. After incubation, we observed a small aliquot of each lysate under 1000x phase-contrast microscopy to confirm the presence of active *Bdellovibrio*, then filtered the lysates and centrifuged the resulting filtrates following the procedure described above to harvest attack-phase *Bdellovibrio*. To prepare genomic DNA from the pelleted *Bdellovibrio* cells, we used the Wizard Genomic DNA purification kit (Promega) following the manufacturer’s instructions beginning with resuspension of the pellet in 600 μL of the kit’s nuclei lysis solution. At the final step, we rehydrated genomic DNA in PCR grade water to avoid inhibition of downstream library preparation or sequencing.

We quantified genomic DNA concentration using a Qubit then diluted with PCR grade water as needed to yield 35 ng/μL. We shipped dilutions on ice to the Rhode Island Genomics and Sequencing Center (RIGSC) for library preparation and sequencing. After shearing genomic DNA using a Covaris S-220 focused ultrasonicator, RIGSC prepared libraries using the PrepX DNA library kit, visualized the libraries on high-sensitivity BioAnalyzer chips, and quantified them using KAPA Illumina quantification kits. RIGSC sequenced libraries on an Illumina MiSeq instrument using 500-cycle v2 chemistry to generate 2 × 250-bp paired-end reads.

We analyzed the raw FASTQ files using breseq 0.35.5 in polymorphism mode (32). We used the default parameter values except for the following: polymorphism-minimum-total-coverage-each-strand 10 and polymorphism-bias-cutoff 0.05. These settings were based on comparisons of different parameter values; for example, we determined that imposing the polymorphism-bias-cutoff, which is off by default, rejected some predicted mutations due to failing Fisher’s exact test for strand bias as applied by *breseq*. Our comparisons showed that base quality did not affect the output under different parameter values, based on the Kolmogorov-Smirnov test for bias in base quality as applied by *breseq*. We also determined that changing polymorphism-reject-indel-homopolymer-length from the default setting of 3 to 5 did not affect the output, therefore we retained the default setting.

To validate *breseq* output obtained using the modified parameter values, we compared the list of mutations identified by *breseq* as present at a frequency of >50% in the evolved *Bdellovibrio* populations with a list of variants detected by a short read analysis workflow using bwa-mem 0.7.13 (33) to align reads and Pilon 1.22 (34) to identify variants occurring in the majority of reads. For four of the evolved *Bdellovibrio* populations, *breseq* and the bwa/Pilon workflow identified the same mutations. For 40D, *breseq* identified an additional mutation not detected by the bwa/Pilon workflow. This mutation was a copy number variant of a four-nucleotide repeat in an intergenic region, which is challenging to reconstruct with short read data. For 40F, *breseq* identified two additional mutations not detected by the bwa/Pilon workflow. One of the mutations was a 189 bp deletion reported at a frequency of 56% by *breseq*. Pilon flagged the region, including this deletion as a local continuity break with no solution. The other mutation was a single-nucleotide substitution reported at a frequency of 58% by *breseq*. Pilon did not report this mutation, but manual examination of the alignment confirmed the *breseq* output. Overall, our comparison of *breseq* output obtained using the modified parameter values and output obtained from the bwa/Pilon workflow showed very good agreement.

### Predatory phenotypes of evolved *Bdellovibrio* populations.

To compare the prey range of evolved *Bdellovibrio* populations to the ancestor *Bdellovibrio* sp. NC01, we made lysates following the procedure described above for genome sequencing, using the −80°C freezer stocks of the *Bdellovibrio* populations at passage 40. Following the double agar overlay protocol described in (3), we tested whether each evolved *Bdellovibrio* population could form plaques on lawns of eight different Gram-negative bacteria. These strains were originally used to test prey range of the ancestor *Bdellovibrio* sp. NC01.

To compare the evolved *Bdellovibrio* populations’ predation efficiency on Pseudomonas to that of the ancestor *Bdellovibrio* sp. NC01, we made lysates following the procedure described above for genome sequencing, using the −80°C freezer stocks of the *Bdellovibrio* populations at passage 40. Following the predation efficiency protocol described above for predatory phenotypes of the ancestor, we quantified the ability of each of the evolved *Bdellovibrio* populations to kill Pseudomonas sp. NC02. We performed t-tests and ANOVA in R.

To compare ancestral and evolved *Bdellovibrio*’s ability to survive starvation, we made lysates following the procedure described above for genome sequencing, using the −80°C freezer stocks of the *Bdellovibrio* populations at passage 40. We then followed the predation efficiency protocol, but we did not include any prey bacteria in the flasks. Instead, we incubated *Bdellovibrio* in HM buffer in the absence of prey for 72 h. We quantified viable *Bdellovibrio* at the beginning and end of the incubation period via plaque counts, then performed ANOVA and *post hoc* analysis in R.

### Data availability.

Sequencing data are available at NCBI under BioProject PRJNA753624 and Sequence Read Archive accession numbers SRR15412329 to SRR15412364 (totaling 36 records).
