# Supplementary material for: Parallel Evolution in Predatory Bdellovibrio sp. NC01 during Long-Term Coculture with a Single Prey Strain
Source: Appl Environ Microbiol. 2023 Jan 4;89(1):e01776-22. doi: 10.1128/aem.01776-22 (PMC9888234; doi:10.1128/aem.01776-22)
Supplement: Supplemental file 1 — Supplemental material. Download aem.01776-22-s0001.pdf, PDF file, 0.1 MB [file aem.01776-22-s0001.pdf]

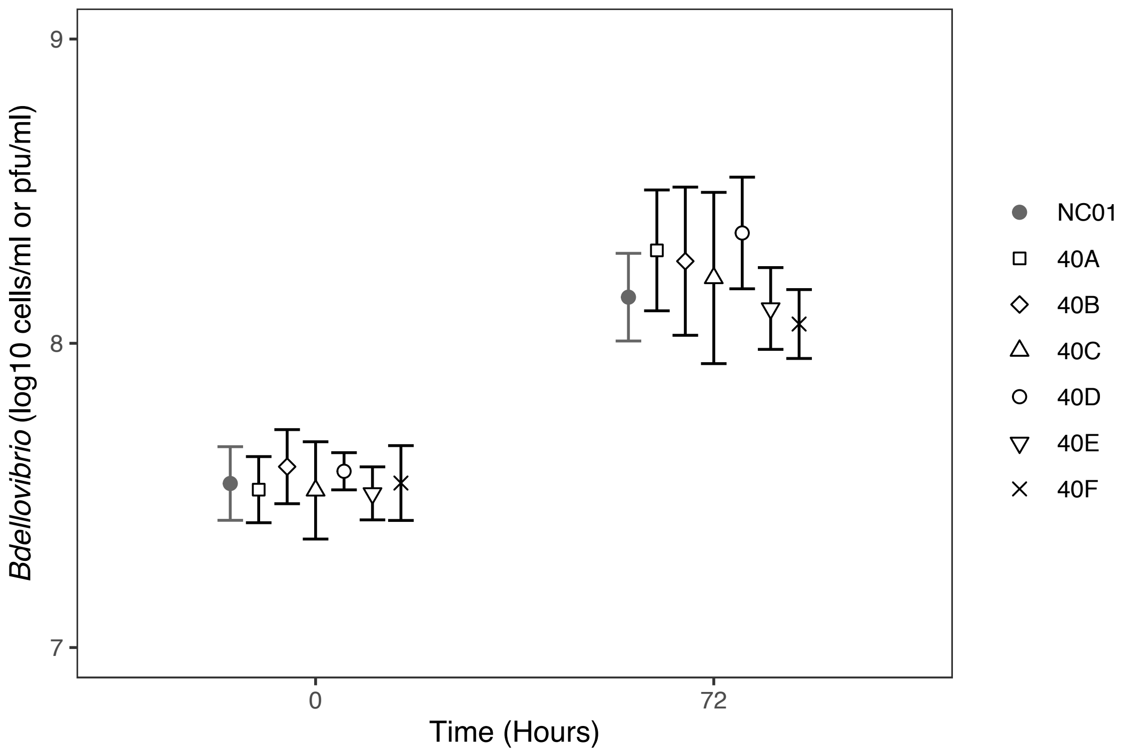


Supplemental Figure 1.

Concentration of *Bdellovibrio* after 72 hours of co-culture with *Pseudomonas* sp. NC02. When conducting the predation efficiency assay to test ancestral and evolved *Bdellovibrio*'s ability to kill *Pseudomonas*, we quantified *Bdellovibrio* at the start (0 h) and end (72 h) of the assay. The data were log transformed and plotted using ggplot in RStudio. Each data point shows the mean and standard deviation for at least four replicates. Analysis of the six evolved *Bdellovibrio* populations at each time point using one-way ANOVA did not detect any statistically significant differences ($p > 0.05$). Comparison of each evolved *Bdellovibrio* population to the ancestor *Bdellovibrio* sp. NC01 at each time point using t-test also did not detect any statistically significant differences ($p > 0.05$).

**Supplemental Table 1. Persistence of new mutations in *Bdellovibrio* populations.**

| | **One** | **Two** | **Three** | **Four** | **Five** | **Six** |
|---|---|---|---|---|---|---|
| **A** | 27 | 4 | 2 | 1 | 0 | 0 |
| **B** | 26 | 7 | 6 | 1 | 0 | 0 |
| **C** | 16 | 8 | 4 | 1 | 1 | 0 |
| **D** | 24 | 10 | 3 | 2 | 0 | 0 |
| **E** | 24 | 9 | 4 | 1 | 0 | 0 |
| **F** | 23 | 4 | 4 | 1 | 2 | 0 |

For each new mutation, we determined whether it was detected in only one of the six time points analyzed or in multiple time points.
